# Flavor Chemical Profiles of Cabernet Sauvignon Wines: Six Vintages from 2013 to 2018 from the Eastern Foothills of the Ningxia Helan Mountains in China

**DOI:** 10.3390/foods11010022

**Published:** 2021-12-23

**Authors:** Xue Zhang, Keqing Wang, Xiaobo Gu, Xiaohan Sun, Gang Jin, Junxiang Zhang, Wen Ma

**Affiliations:** 1 School of Food & Wine, Ningxia University, Yinchuan 750021, China; zhangxue19950304@163.com (X.Z.); wangkeqinghe@163.com (K.W.); 13598070297@163.com (X.G.); Caesar7941@163.com (X.S.); gjinwine@hotmail.com (G.J.); zhangjunxiang@126.com (J.Z.); 2 Engineering Research Center of Grape and Wine, Ministry of Education, Yinchuan 750021, China; 3 School of Agriculture, Ningxia University, Yinchuan 750021, China

**Keywords:** Cabernet Sauvignon, volatile compounds, phenolic profile, Ningxia, wine regionality

## Abstract

The eastern foothills of the Helan Mountains in the Ningxia region (Ningxia), is a Chinese wine-producing region, where Cabernet Sauvignon is the main grape cultivar; however, little compositional or flavor information has been reported on Ningxia wines. Oenological parameters, volatile profiles, and phenolic profiles were determined for 98 Ningxia Cabernet Sauvignon wines from the 2013–2018 vintages, as well as 16 from Bordeaux and California, for comparison. Ningxia wines were characterized by high ethanol, low acidity, and high anthocyanin contents. Multivariate analysis revealed that citronellol and 12 characteristic phenolic compounds distinguish Ningxia wines from Bordeaux and California wines. The concentrations of most phenolic compounds were highest in the 2018 Ningxia vintage and decreased with the age of the vintage. To our knowledge, this is the first extensive regionality study on red wines from the Ningxia region.

## 1. Introduction

Grape (*Vitis vinifera*) Wine is an alcoholic beverage with a complex flavor, related to its composition of alcohol, sugar, acid, phenolics, and aroma volatiles [1]. The flavor is influenced by the grape cultivar, fermentation conditions, and aging process, as well as “terroir”, the growing conditions of the grapes, including soil, geography, climate, and viticultural practices [2,3], characteristic of each growing region [4]. The grape composition is significantly affected by terroir, along with climate/soil/plant interactions [5,6]. For example, grapes growing in a climate with a large diurnal temperature difference have higher sugar concentrations, higher photosynthetic rates, and improved color and taste, under otherwise similar environmental conditions [7], which influences the quality of the final wine product.

The term terroir originated in France, is related to the reputation of each producing region and their distinguishing characteristics, and adds economic value, through the “character” of the wine, which appeals to consumers [6]. Wines from different regions and vintages can be characterized and distinguished by “fingerprinting” of aroma volatiles, phenolic compounds, minerals, amino acids, and other components [8,9,10,11,12]. For example, Sauvignon blanc wines from New Zealand, France, and Austria were analyzed by sensory evaluation, gas chromatography (GC), and multivariate statistical analysis (ANOVA, CA, PLS-DA, etc.) [8], demonstrating that wines of different geographical origin had clearly distinguishable profiles. New Zealand wines were characterized by “green” flavor notes, such as green capsicum, herbaceous and grassy, Austrian wines by the fruity/boxwood compound 4-mercapto-4-methylpentan-2-one (4MMP), and French wines by other compounds (e.g., benzaldehyde). Regional differences between Sangiovese wines from California and Italy could be distinguished, based on chemical profiling of aroma volatiles, pigments, polyphenols, and minerals, by multivariate analysis [9]. A similar approach to Cabernet Sauvignon wines from Xinjiang (China) demonstrated that contents of terpenes and phenylalanine derivatives of the wines from northern Xinjiang were higher than those from the south [10]. The phenolic profiles of Cabernet Sauvignon wines from five wine-growing regions in China (Helan mountains of Ningxia, Qilian of Gansu, Changli of Hebei, Yantai of Shandong, and Deqin of Yunnan), were related to the terroir of the growing region [2]; a similar terroir resulted in a similar polyphenolic profile. Another allied analysis found the content of phenols and aroma substances was affected by altitude, phenolic, and sensory profiles could achieve the separation of Malbec wines from different regions of Mendoza, Argentina, which evinced the effects of “terroir” [13]. Determination of polyphenolic or mineral fingerprints was able to distinguish between vintages and growing regions of Argentinian Malbec wines [6] and Australian Pinot noir wines [11], respectively. In recent years, various studies on origin in various producing areas are still as a topic of great interest ongoing, such as elemental and phenolic analysis of Italian (Veneto, Umbria, and Friuli) wines, metabolomic profiles of Spanish (Rioja and Priorat) red wines, and color and volatile analyses of Australian (Adelaide Hills, Yarra Valley, Mornington Peninsula, Northern and Southern Tasmania) wines, etc. [14,15,16,17]. Meanwhile, the study about wines of different grape varieties and origins, yeast strains, and spontaneous fermentation also strongly indicated that volatile chemical differences between wines were mainly driven by grape origin [18].

The eastern foothills of the Helan Mountains in the Ningxia region (Ningxia) is an emerging Chinese wine-producing region, with a latitude of 37°43′–39°23′, a longitude of 105°45′–106°47′, and a temperate continental climate. Ningxia is a relatively new wine-producing region; grape growing started in 1982 [19], and it applied for protection of geographical indication in 2004 (GB/T 19504-2004). Cabernet Sauvignon is the main red grape cultivar in Ningxia, because of its high quality and adaptability to growth in different environments. Many studies on the relationship between the terroir and wine flavor characteristics from winemaking regions worldwide have been reported, but that of Ningxia, specifically the identification of characteristic flavors attributable to the region, has not been reported.

In this study, the following three types of indicators were analyzed: (1) oenological parameters; (2) volatile compounds; (3) phenolic profiles. They were determined to construct a flavor fingerprint for 98 Cabernet Sauvignon wines, from the six sub-regions of the Ningxia region, for comparison with the fingerprints of 16 Cabernet Sauvignon wines from California and Bordeaux. Two common analysis methods were used for aroma volatiles (headspace–solid-phase extraction–gas chromatography–mass spectrometry (HS–SPME–GC–MS)) and polyphenols (ultra-high-performance liquid chromatography-quadrupole–time-of-flight mass spectrometry; UHPLC-Q–ToF), and the results were analyzed by analysis of variance (ANOVA), principal components analysis (PCA), and hierarchical cluster analysis (HCA). The aim was to relate flavor profiles to geographical locations (GLs) in the Ningxia region, characterize different vintages, and inform consumers about the unique character of Ningxia wines.

## 2. Materials and Methods

### 2.1. Wine Samples

**Figure 1:**
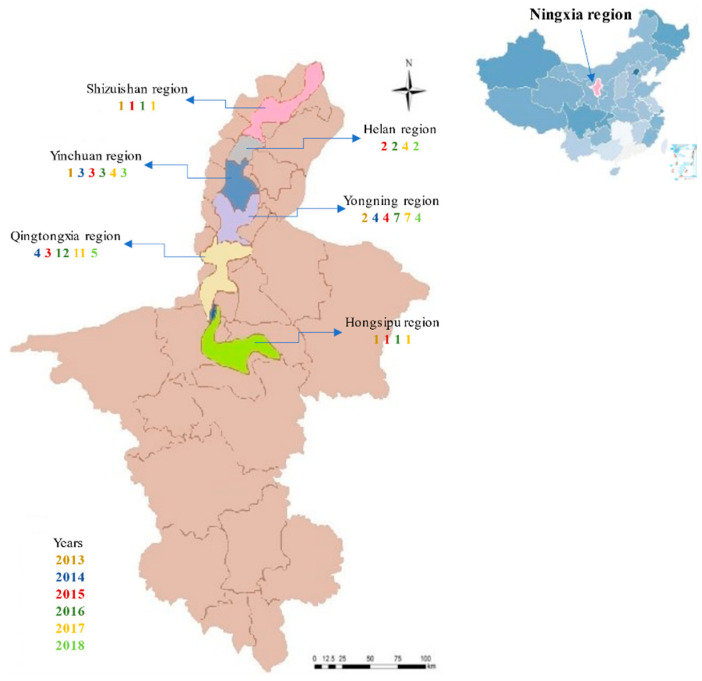
Distribution map of wine samples of six vintages (2013–2018), from the six sub-regions from the Ningxia region of China. (The colored numbers represent the number of samples from each vintage that were collected from each sub-region.).

In total, 98 commercial wines from the eastern foothills of the Ningxia Helan Mountains, of vintages 2013–2018 were collected; the wines were chosen from six different sub-regions (Hongsipu, Qingtongxia, Yongning, Yinchuan, Helan, and Shizuishan; Figure 1). All of the wines were made from pure *Vitis vinifera* L. Cabernet Sauvignon, or from less than 10% of other grape varieties.

For comparison, 16 Cabernet Sauvignon or blended wines from the 4 older vintages (2014–2017) from 2 Cabernet Sauvignon wine0producing regions—Bordeaux and California—were also collected. All of the wines were sourced from commercial producers (Table S1). All the analyses were carried out in the autumn of 2019.

### 2.2. Chemicals and Standards

NaOH, phenolphthalein, tartaric acid, Na_2_CO_3_, and gallic acid were from Tianjin Damao Chemical Reagent Factory (Tianjin, China); Folin–Ciocalteu phenol assay reagent was from Shanghai Ruiyong Biological Technology Co., Ltd. (Shanghai, China); HCl was from Tianjin Miou Reagent Co., Ltd. (Tianjin, China); ethanol, NaHSO_3_, NaCl were from Sinopharm Chemical Reagent Co., Ltd. (Shanghai, China); methanol and formic acid were from Thermo Fisher Scientific Co., Ltd. (Shanghai, China); LC–MS grade water was from Honeywell International Co., Ltd. (Morris Plains, NJ, USA).

Aroma volatile standards, isoamyl alcohol, isoamyl acetate ≥99.7%, acetoin ≥98.0%, and nonanal ≥98.0% were from Sigma Aldrich (St. Louis, MO, USA); 3inalool ≥96.0%, 1-octanol ≥99.7%, methionol ≥99.0%, and 4-methyl-2-pentanol ≥98.0% were from Tokyo Chemical Industry (Tokyo, Japan); phenolic standards, (+)-catechin 99.2%, (−)-epicatechin 99.7%, (−)-epicatechin gallate 98.1%, 3,4-dihydroxybenzoic acid 99.9%, and gallic acid 90.9% were from the National Institutes for Food and Drug Control (Beijing, China); gallocatechin 98.54% and epigallocatechin 99.77% were from Chengdu Biopurify Phytochemicals Ltd. (Chengdu, China); quercetin dihydrate ≥98.0%, myricetin ≥98.0%, isorhamnetin ≥98.0%, and syringic acid ≥98.0% were from Solarbio Science and Technology Co., Ltd. (Beijing, China); caffeic acid 98.0% was from Yuanye Bio-Technology Co., Ltd. (Shanghai, China).

### 2.3. Oenological Parameter Determination

The pH, titratable acidity, and alcohol content were measured according to the National Standard of the People’s Republic of China (GB/T15038-2006, 2006) [20]. The Folin–Ciocalteu method was used to determine total phenols [21], the Bate–Smith method was used to determine total tannins [22], and the SO_2_ bleaching method was used to determine total anthocyanins [23].

### 2.4. Aroma Volatile Analysis by HS–SPME–GC–MS

Volatile compounds were analyzed according to the method of Wilkinson et al. [24], with minor modifications.

Solid-phase microextraction (SPME): An aliquot of wine (5 mL) was added to a 20 mL glass headspace vial containing NaCl (1.5 g, Guangnuo Chemical Technology, Shanghai, China); then, internal standard (4-methyl-2-pentanol, 10 μL, 1008.3 mg/L in ethanol) was added to a final concentration of 2.01 mg/L. The mixture was agitated on an orbital shaker at 250 r/min and 40 °C for 5 min. The SPME fiber (50/30 μm divinylbenzene/carboxen/polydimethylsiloxane (DVB/CAR/PDMS), length 1 cm Supelco, Bellefonte, PA, USA) was aged at 250 °C for 10 min, cooled, and then added to the sample vial and agitated at 40 °C for an additional 30 min

GC–MS analysis: The GC–MS analysis was performed on an Agilent 7890B gas chromatograph (Agilent, Santa Clara, CA, USA), fitted with a Gerstel PAL RSI 85 autosampler (CTC Analytics AG, Zwingen, Switzerland) and an Agilent 7000D mass-selective detector. Thermal desorption of analytes from the SPME fiber was achieved by splitless injection (straight glass liner, 0.75 mm i.d.) at 240 °C for 10 min. The column oven temperature program was as follows: initial temperature 40 °C, held for 5.0 min, increased to 97 °C at 3 °C/min, then to 120 °C at 2 °C/min, then to 150 °C at 3 °C/min, then to 220 °C at 8 °C/min, then held for 10 min. The MS detector was operated in scan mode (mass range 40−300 *m*/*z*), the transfer line to the MS system was maintained at 230 °C, the interface between the GC and the MS was held at 240 °C, and the electron impact source operated at 70 eV.

Identification and quantification of volatile compounds: A total of 47 volatile compounds were identified in the wines (Table S2 and Figure S1) using experimentally obtained Kovats retention indices (RI), with the combination of C8–C40 alkane standards and mass spectra in the NIST MS library. Aroma volatile standard mixtures were prepared in a red wine sample. Standard concentrations were selected to bracket the concentrations of each individual compound as closely as possible. All standards were analyzed in duplicate. Any compound for which the reference standard was not available was quantitated based on the standard with the closest structure available. In total, 19 volatile compounds were quantified by standard concentrations and were used for data analysis.

### 2.5. Phenolic Compound Analysis by UHPLC-Q–ToF

All wines were analyzed for monomeric phenolics using an UHPLC-ESI-Q–ToF (Agilent 1290II-6546, Santa Clara, CA, USA) equipped with a dual Jet Stream ESI source operated in negative ion mode. A C18 UHPLC column (2.1 × 100 mm, 1.8 μm, Agilent Zorbax eclipse plus, Santa Clara, CA, USA) was used for separation, as described previously [25], with minor modifications.

UHPLC conditions: The mobile phases were water (A) and methanol (B), both containing 0.1% formic acid. The flow rate was 0.4 mL/min, and the column temperature was 35 °C. The initial mobile phase was 10% B; then, the percentage of B was ramped linearly to 20% B at 4 min, 25% B at 8 min, 36% B at 12 min, 50% B at 14 min, 95% B at 14.6 min, held until 17.6 min, back to 10% B at 18.2 min, and held until 20 min.

ESI-Q–ToF–MS analysis conditions: MS analysis was performed with an extended dynamic range of 2 GHz (*m*/*z* 3200 Th). The nebulizer pressure and flow rates were 25 psi and 9 L/min; drying gas temperature 300 °C; sheath gas flow and temperature 11 L/min and 350 °C; fragmentation, skimmer, OCT, and capillary voltages 150, 65, 750, and 4000 V, respectively. All analyses were performed in negative mode. The collision energies for MS/MS analysis were 5, 10, or 20 V for different compounds. Mass calibration was achieved by infusion of a TOF ESI Tune Mix solution (standard mix G1969-85,000, Supelco Inc., Bellefonte, PA, USA) containing compounds with negative ionization *m*/*z* values of 112.985587, 301.998139, 601.978977, 1033.988109, 1333.968947, 1633.949786, 1933.930624, 2233.911463, 2533.892301, and 2833.873139, and had a residual error for the expected masses of ±0.3 ppm. The data analysis was performed on MassHunter Qualitative Analysis software (Agilent, Santa Clara, CA, USA).

Identification and quantification of phenolic compounds: Overall, 20 phenolic compounds were identified in wines and identified by matching their retention times with those of authentic standards (Table S3 and Figure S2). Standard concentrations were selected to bracket the concentrations of each individual compound as closely as possible, and all standards were analyzed in duplicate. Except for gallocatechin, 19 compounds quantified by standard concentrations were used for data analysis. Phenolic compounds standard mixtures were prepared in methanol aqueous solution that 1:1 (*v*/*v*).

### 2.6. Statistical Analysis

Linear regression and statistical calculations were performed using Microsoft Excel. An analysis of variance (ANOVA), followed by the least significant difference (LSD) post hoc test, was carried out using R3.6.3 (R Core Team, Vienna, Austria). Differences were considered significant if the p-value was below 0.05. PCA was used to explore the relationships between regions and samples by these compounds with significance. HCA was used to explore the relationships between samples and regions, and samples and years. These analyses were performed on the agricolae, pheatmap, and FactoMineR packages in Rstudio (R Core Team, Vienna, Austria).

## 3. Results and Discussion

### 3.1. Classification of Wines by Geographical Origin and Vintage Based on Oenological Parameters

Oenological parameters, such as alcohol content, titratable acidity, pH, total phenols, total anthocyanins, and total tannin, are essential parameters for red wine quality [3,26,27,28]. Differences in oenological parameters (alcohol content, titratable acidity, pH, total phenols, total anthocyanins, and total tannin) between the six sub-regions of Ningxia were explored by ANOVA; rarely significant differences were found (Table 1).

Differences in oenological parameters between wines from Ningxia, California, and Bordeaux were also explored, and five out of six had significant differences (Table 1). The alcohol content of Ningxia (13.76% *v*/*v*) wine was significantly higher than that of Bordeaux (12.93% *v*/*v*). The total acidity of Ningxia (5.81 g/L) wine was significantly lower than that of Bordeaux (7.29 g/L). The total anthocyanin content of California (0.20 g/L) and Ningxia (0.17 g/L) wines was significantly higher than that of Bordeaux (0.09 g/L). Since the ethanol content of wine depends on the sugar concentration in the fermentation, wines from warm, dry regions tend to have higher ethanol concentrations than those from cool, wet regions [29]. The high alcohol and anthocyanin content of Ningxia wines appears to be related to the inland location of the Helan Mountains, which have a typical temperate, semi-arid continental monsoon climate. The eastern foothills have a large day/night temperature difference, high annual sunshine hours, and a short monsoon season, which promote the accumulation of high sugar and anthocyanin contents in the grapes. The large day/night temperature difference results in the rapid accumulation of sugar and low acidity. The Ningxia climate, therefore, produces wines with high alcohol and anthocyanin content but low acidity. California (known as the Sunshine Coast) has very high annual sunshine hours, so the wine anthocyanin content is higher than that of Ningxia. There was no significant difference in pH, total phenols, or total tannins between the three regions (Table 1).

**Figure 2:**
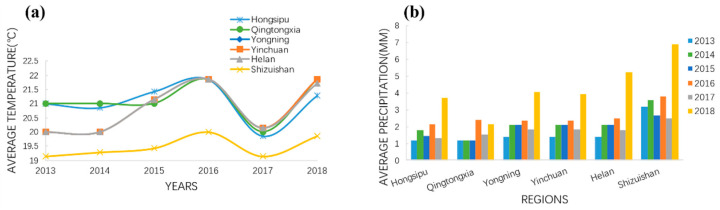
Average temperature (**a**) and precipitation (**b**) records from March to September 2013–2018 in the six sub-regions of Ningxia.

Differences in oenological parameters between the six Ningxia vintages (2013, 2014, 2015, 2016, 2017, and 2018) were explored (Table 2). Alcohol and anthocyanin content followed an overall downward trend as aging time increased. The alcohol content of the recent vintages, from 2016 (13.93% *v*/*v*), 2017 (13.87% *v*/*v*), and 2018 (13.93% *v*/*v*) was significantly higher than that from 2013 (12.92% *v*/*v*). One explanation for this could be that a warmer climate increases the alcohol content of wines from a given region [30,31]. Climate change has resulted in a global temperature rise, which promotes an increased accumulation of sugar. The weather records for Ningxia, from March to September 2013–2018 (Table S4 and Figure 2), show that the average temperature and precipitation have been increasing over this period, except for a decrease in 2017. Another explanation for decreased alcohol content in older vintages could be volatility and chemical reactions such as oxidation and esterification, during the aging process. The anthocyanin content of the near vintage wine of 2018(0.24 g/L) was significantly higher than that of the long-aged wine of 2013 (0.05 g/L), 2014(0.08 g/L), 2015 (0.13 g/L), and 2016 (0.15 g/L). This may be due to the polymerization and precipitation during wine aging.

The anthocyanin content of the recent 2018 vintage (0.24 g/L) was markedly higher than that of the older vintages from 2013 (0.05 g/L), 2014 (0.08 g/L), 2015 (0.13 g/L), and 2016 (0.15 g/L). Anthocyanins may undergo chemical reactions, such as cyclization, oxidation, and polymerization, to form various anthocyanin derivatives, resulting in decreased anthocyanin content [32].

### 3.2. Classification of Wines by Geographical Origin Based on Volatile Compounds

Volatile compounds only account for 2% of the wine composition but make a major contribution to its flavor; they are an important indicator of the flavor characteristics of a wine. The number of volatile compounds detected in wine ranged from 400 (1969) to more than 1300 (1983), and tens of thousands of chemical signals in recent vintages [29]. In this study, 47 volatile compounds were identified (Table S2). In total, 19 volatile compounds were quantified by the standard concentrations, including esters (isoamyl acetate, ethyl hexanoate, ethyl caprylate, ethyl caprate, diethyl succinate); alcohols (isobutanol, isoamyl alcohol, 1-hexanol, benzyl alcohol, 1-decanol, methionol, and phenylethyl alcohol); terpenoids (p-cymene, and citronellol); aldehydes and ketones (acetoin, nonanal, furfural, decanal, and benzaldehyde). After ANOVA, compounds with significant differences were used as variables in PCA.

**Figure 3:**
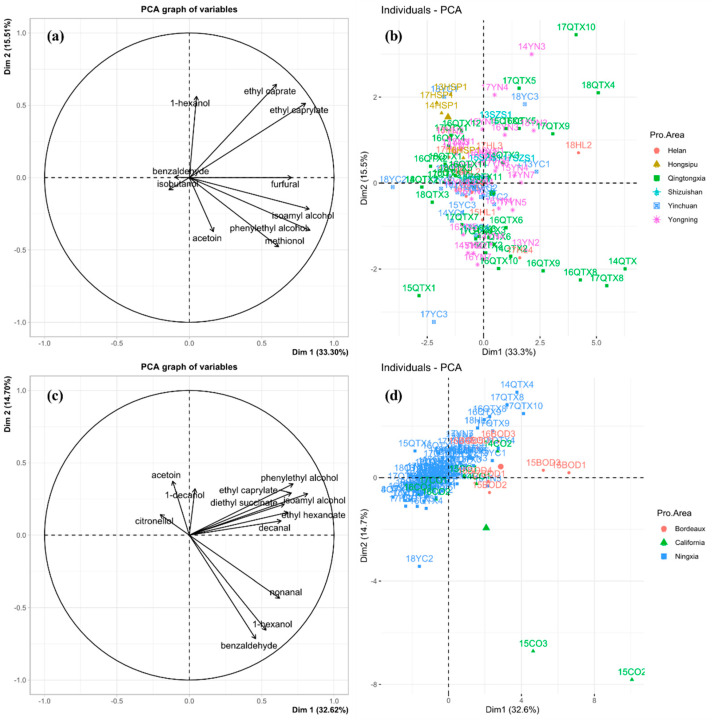
PCA (principal components analysis) scores plot for wines based on volatile compounds of six sub-regions of Ningxia (**a**) and three wine-producing regions (**c**). PCA loadings plot of volatile compounds of six sub-regions of Ningxia (**b**) and three wine-producing regions (**d**).

Principal components analysis (PCA) was used to display similarities and differences in volatile composition with significance between samples. The vectors in the variables plot (Figure 3a,c) show the relative loadings of the most variable volatile compounds. The individual plot (Figure 3b,d) shows the similarities and differences between the wine samples. There were fewer volatile compounds with significant differences in concentration among the 6 Ningxia sub-regions (10 volatile compounds) than between Ningxia, California, and Bordeaux (12 volatile compounds).

The first (PC1, 33.3%) and second (PC2, 15.5%) principal components (Figure 3a,b), explained 48.8% of the total variance between the six Ningxia sub-regions. The wine samples from five of the sub-regions (Helan, Qingtongxia, Yinchuan, Yongning, and Shizuishan) were grouped together, indicating that their volatile compound profiles were similar, although those from Qingtongxia were much more scattered, i.e., more variable, than those of the other regions (Figure 3b). However, wines from the Hongsipu sub-region were moderately separated from the other five sub-regions (Figure 3a); the 10 volatile compounds and Hongsipu wines had opposite trend vectors in PC1, indicating that the volatile compound contents were negatively correlated with the Hongsipu wines. Overall, however, the Hongsipu wines had more similarities than differences, compared with those of the other sub-regions, and the differences may result from Hongsipu being the furthest south, with the warmest and driest climate (Figure 2).

Differences in volatile compounds between wines from Ningxia, California, and Bordeaux were compared by PCA (Figure 3c,d), to investigate regional effects on the aroma profile. The first (PC1, 32.6%) and second (PC2, 14.7%) components explained 47.3% of the total variance. The wines were divided into three regional groups in the individual plots, with the Ningxia group on the upper left side and the California and Bordeaux groups on the bottom right side (Figure 3d). The Ningxia wines were relatively closely grouped. Except for nonanal, 1-hexanol, and benzaldehyde, all the volatile compounds in quadrants one, two, and three of the variables plots (Figure 3c) were all present in Ningxia wines, and quadrant two contained only compounds in Ningxia wines. Citronellol and acetoin in quadrant two are the characteristic compounds that distinguish Ningxia wines from those of Bordeaux and California. Bordeaux wines were mainly distributed in quadrant one and were characterized by phenylethyl alcohol, ethyl caprylate, isoamyl alcohol, butanedioic acid, diethyl ester, ethyl hexanoate, and decanal. California wines were relatively scattered and characterized by benzaldehyde, nonanal, and 1-hexanol in quadrant four. In summary, the aroma profiles of wines from the six Ningxia sub-regions were similar but clearly distinct from those of California and Bordeaux. PCA analysis of aroma profiles can, therefore, clearly distinguish between Cabernet sauvignon wines from the three producing regions. Additionally, it can be seen from Figure 3d that in this study, region is the primary indicator affecting wine volatile compounds. Although vintage is an important indicator affecting wine quality, the distribution of samples was more concentrated in the region. This may benefit from the unique geographical environment of the producing region.

**Figure 4:**
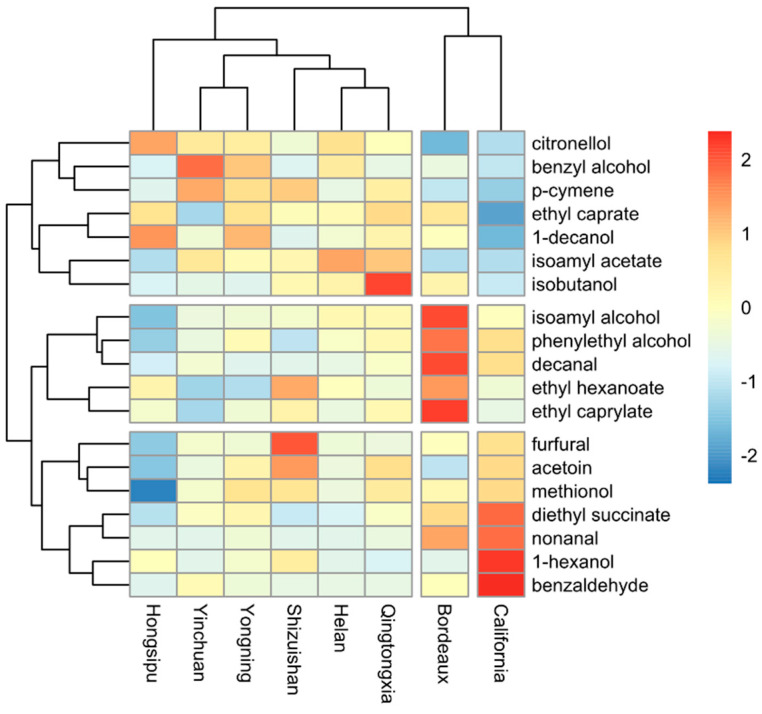
HCA (hierarchical cluster analysis) of wines based on volatile compounds from the six sub-regions of Ningxia, Bordeaux, and California.

The volatile compounds from the six consecutive Ningxia vintages (2013–2018) were analyzed using the HCA, to compare differences between the Ningxia sub-regions, Bordeaux, and California. The 19 volatile compounds were clustered into 3 groups in the vertical clustering tree, and the wines from Ningxia (all 6 sub-regions), Bordeaux, and California formed 3 groups in the longitudinal clustering tree (Figure 4). Ningxia wines were mainly characterized by citronellol, benzyl alcohol, ethyl caprate, p-cymene, 1-decanol, isoamyl alcohol, isoamyl acetate, and isobutanol. Bordeaux wines were mainly characterized by isoamyl alcohol, phenylethyl alcohol, decanal, ethyl hexanoate, and ethyl caprylate. California wines were mainly characterized by furfural, acetoin, methionol, butanedioic acid, diethyl ester, nonanal, 1-hexanol, and benzaldehyde. In addition, the six Ningxia sub-regions were partially separated into sub-sets; Yinchuan and Yongning had very similar aroma profiles, as did Helan and Qingtongxia, and both pairs formed the closest subsets. Hongsipu had a distinct aroma profile from the other sub-regions and formed a separate sub-group. The differences between the Ningxia, California, and Bordeaux regions were very clear; their volatile profiles were markedly different. This undoubtedly benefits from the unique geographical environment of the production area, although there may be an impact of vintage on the volatile compounds of wine.

### 3.3. Classification of Wines by Geographical Origin and Vintage Based on Phenolic Compounds

Phenolic compounds are important indicators of the taste characteristics of wine, such as astringency, bitterness, and mouth-feel, and they are present both as monomers and oligomers [26,33]. In total, 19 phenolic compounds were quantified by standard curve. Including flavanols (epicatechin gallate, epigallocatechin, catechin, and epicatechin), procyanidin oligomers (dimer1, dimer2, dimer3, dimer4, dimer5, dimer6, trimer1, and trimer2), flavonols (myricetin, isorhamnetin, and quercetin), and phenolic acids (syringic, caffeic, gallic, and protocatechuic acids). After ANOVA, compounds with significant differences were used as variables in PCA.

**Figure 5:**
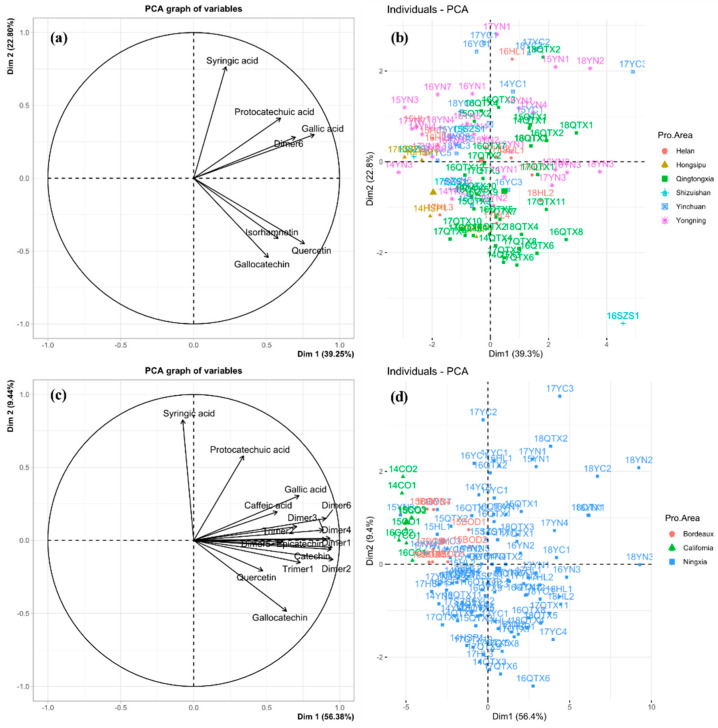
PCA (principal components analysis) scores plot for wines based on phenolic compounds of six sub-regions of Ningxia (**a**) and three wine-producing regions (**c**). PCA loadings plot of volatile compounds of six sub-regions of Ningxia (**b**) and three wine-producing regions (**d**).

PCA was used to display similarities and differences in phenolic composition with significance between samples. The vectors in the variables plot (Figure 5a) show the relative loadings of the most variable phenolic compounds. The individual plot (Figure 5b) shows the similarities and differences between the wine samples. There were fewer phenolic compounds with significant differences between the six Ningxia sub-regions (seven phenolic compounds) than between Ningxia, California, and Bordeaux (sixteen phenolic compounds).

In the PCA plot of phenolic compounds from the 98 wines from the 6 Ningxia sub-regions (Figure 5a), the first (PC1, 39.2%) and second (PC2, 22.8%) components explained 62.0% of the total variance in the data set. The wine samples from the six Ningxia sub-regions formed a loose group (Figure 5b) and were evenly distributed over the four quadrants. The seven characteristic phenolic compounds of the six sub-regions formed similar profiles in wines from all the regions.

In the PCA variables plot of phenolic compounds from the 114 wines from Ningxia, Bordeaux, and California (Figure 5c), the first (PC1, 56.4%) and second (PC2, 9.4%) components explained 65.8% of the total variance in the data set. The wines were divided into three groups, with the Ningxia wines mainly in quadrants one, three, and four, and those from California and Bordeaux in quadrant two (Figure 5d). The contribution of the 16 characteristic phenolic compounds (Figure 5c) to Ningxia wines was significantly higher than those from Bordeaux and California. In the Bordeaux and California wines, these compounds had opposite trend vectors in PC1, i.e., they were negatively correlated with the Bordeaux and California wines. The regional variation in phenolic compound profiles was similar to that of the volatile compound profiles; those of the six Ningxia sub-regions were similar, whereas those of the Ningxia, California, and Bordeaux regions were markedly different. Additionally, it can be seen from Figure 5d that in this study, a result similar to volatile compounds, distribution of region is the primary indicator affecting phenolic compounds. The distribution of samples was more concentrated in the region rather than vintage.

**Figure 6:**
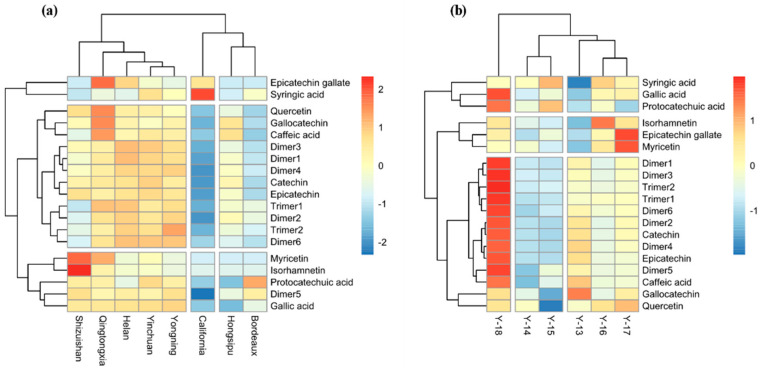
HCA (hierarchical cluster analysis) of wines based on phenolic c ompounds from the six sub-regions of Ningxia, Bordeaux, and California (**a**) wines from the six vintages (2013–2018) from the Ningxia region of China (**b**).

The phenolic compounds from the 114 wines of the 6 consecutive Ningxia vintages (2013–2018) were analyzed using hierarchical cluster analysis (HCA) to compare differences between the Ningxia sub-regions, Bordeaux, and California (Figure 6a). The 19 phenolic compounds were clustered into 3 groups in the vertical clustering tree, and the wines from Ningxia, Bordeaux, and California formed 3 groups in the longitudinal clustering tree (Figure 6a), with the exception of Hongsipu, which had a phenolic profile similar to that of Bordeaux wines and distinct from the other Ningxia wines. Ningxia wines were mostly characterized by 12 phenolic compounds (quercetin, gallocatechin, caffeic acid, catechin, epicatechin, procyanidin oligomers, dimer1, dimer2, dimer3, dimer4, dimer6, trimer1, and trimer2. Bordeaux wines were mostly characterized by protocatechuic acid and dimer5. California wines were mostly characterized by epicatechin gallate and syringic acid. Previous studies have shown that sufficient light and colder weather contribute to the accumulation of some phenols [13]. The high content of most monomeric phenols in Ningxia wine may also be caused by sufficient light and a large temperature difference between day and night in Ningxia. In addition, Yinchuan and Yongning had very similar phenolic profiles and formed a subset with Helan, which had a slightly different profile. The Ningxia sub-regions are adjacent, with the same longitude and in the order Shizuishan, Helan, Yinchuan, Yongning, Qingtongxia, and Hongsipu, from north to south, over a distance of ~300 km (Figure 1). The phenolic profiles of these five sub-regions had generally similar phenolic profiles and were grouped relatively closely. The exception was Hongsipu, which had a relatively low phenolic content, compared with the other sub-regions. Most of the phenolic compounds in the Hongsipu wines were significantly higher than Bordeaux and California wines, but the phenolic profile was most similar to that of Bordeaux. The differences in Hongsipu wines may be related to it being the southernmost region and having a warmer, dryer climate than the other regions (Figure 2). The differences between the Ningxia, California, and Bordeaux regions were very clear; their phenolic profiles were markedly different.

The phenolic compounds from the six consecutive Ningxia vintages (2013–2018) were analyzed using hierarchical cluster analysis (HCA) to compare differences between them (Figure 6b). The 19 phenolic compounds were clustered into 3 groups in the vertical clustering tree, and the wines from the 6 Ningxia vintages formed 3 groups in the longitudinal clustering tree. The phenolic profiles of the 2014 and 2015 vintages, and the 2016 and 2017 vintages were very similar, forming two groups, which were closely related to each other and the 2013 vintage. The phenolic profile of the 2018 vintage was prominent and formed a separate group. The 2018 vintage had the highest overall phenolic content, with 13 identified phenolic compounds (caffeic acid, catechin, epicatechin, gallic acid, protocatechuic acid, dimer1, dimer2, dimer3, dimer4, dimer5, dimer6, trimer1, and trimer2). This may be due to the relatively new vintage in 2018, sufficiently accumulated temperature, and abundant precipitation, compared with other years. The content of most phenolic compounds in the most aged vintages of 2014 and 2015 was significantly lower than the others, especially catechin, epicatechin, quercetin, dimer1, dimer2, dimer3, dimer4, dimer5, dimer6, trimer1, and trimer2. The phenolic content of the 2016 and 2017 vintages was lower than the 2018 vintage and phenolic content followed a downward trend from 2018 to 2013. The content of gallic acid, isorhamnetin, epicatechin gallate, and myricetin in 2013 wine was the lowest of the vintages, followed by 2014 and 2015. The content of most individual phenolic compounds decreased markedly with increasing wine age, as for the anthocyanin content (Table 2).

## 4. Conclusions

In conclusion, the aroma volatile and polyphenolic profiles of Cabernet Sauvignon wines from the six sub-regions of the eastern foothills of the Helan Mountains in the Ningxia region of China were similar but distinct from those of wines from Bordeaux and California. Multivariate analysis of the aroma volatile and polyphenolic profiles could clearly distinguish wines from the three main producing regions studied; the differentiating compounds, which were more abundant in Ningxia wines, were citronellol and 12 phenolic compounds (quercetin, gallocatechin, caffeic acid, catechin, epicatechin, procyanidin dimer1, dimer2, dimer3, dimer4, dimer6, trimer1, and trimer2). The phenolic profiles of the 2014 and 2015, and the 2016 and 2017 vintages were relatively similar, respectively. The phenolic content was the main distinguishing factor between the recent 2018 vintage and the older 2013–2017 vintages. The content of most phenolic compounds, such as caffeic acid, catechin, and epicatechin, was highest in the 2018 vintage. The content of anthocyanins and most phenolic compounds decreased as the wines aged.

## Figures and Tables

**Table 1:** Oenological parameters from the six sub-regions of Ningxia, California, and Bordeaux. (*p* < 0.05).

Name	Helan	Hongsipu	Qingtongxia	Yinchuan	Yongning	Shizuishan	Ningxia	California	Bordeaux
Alcohol content (*v*/*v*, %)	14.18 ± 0.55 ^a^	13.45 ± 1.42 ^ab^	14.04 ± 0.77 ^a^	13.64 ± 0.97 ^ab^	13.30 ± 0.88 ^b^	13.89 ± 0.36 ^ab^	13.76 ± 0.89 ^a^	13.45 ± 0.92 ^ab^	12.93 ± 0.41 ^b^
Titratable acidity (g/L Tartaric acid)	5.72 ± 0.81 ^a^	5.60 ± 0.37 ^a^	5.77 ± 0.67 ^a^	5.70 ± 0.57 ^a^	5.93 ± 0.66 ^a^	6.28 ± 0.31 ^a^	5.81 ± 0.65 ^b^	6.50 ± 0.53 ^ab^	7.29 ± 3.39 ^a^
pH	3.70 ± 0.11 ^a^	3.71 ± 0.04 ^a^	3.73 ± 0.17 ^a^	3.75 ± 0.14 ^a^	3.67 ± 0.15 ^a^	3.72 ± 0.11 ^a^	3.71 ± 0.15 ^a^	3.70 ± 0.16 ^a^	3.75 ± 0.12 ^a^
Total phenols (g/L, gallic acid)	2.93 ± 0.46 ^a^	2.49 ± 0.41 ^a^	2.79 ± 0.66 ^a^	3.00 ± 0.49 ^a^	2.77 ± 0.63 ^a^	2.91 ± 0.42 ^a^	2.83 ± 0.59 ^a^	2.62 ± 0.32 ^a^	2.99 ± 0.34 ^a^
Total anthocyanins (g/L)	0.20 ± 0.08 ^a^	0.16 ± 0.16 ^a^	0.16 ± 0.08 ^a^	0.17 ± 0.12 ^a^	0.15 ± 0.08 ^a^	0.14 ± 0.07 ^a^	0.17 ± 0.09 ^a^	0.20 ± 0.1 2 ^a^	0.09 ± 0.05 ^b^
Total tannin (g/L)	3.18 ± 0.37 ^a^	2.95 ± 0.85 ^a^	3.38 ± 0.98 ^a^	3.21 ± 0.64 ^a^	3.19 ± 0.62 ^a^	3.13 ± 0.57 ^a^	3.25 ± 0.75 ^a^	3.32 ± 0.74 ^a^	3.64 ± 0.52 ^a^

Note: the six sub-regions of the eastern foothills of Helan Mountains and the three regions of Ningxia, California, and Bordeaux were compared separately; Different Latin letters indicate significant differences according to the HSD Tukey test (*p* < 0.05).

**Table 2:** Oenological parameters of red wine from the six vintages (2013–2018) in the Ningxia region. (*p* < 0.05).

Name	2013	2014	2015	2016	2017	2018
Alcohol content (%, *v*/*v*)	12.92 ± 1.04 ^b^	13.36 ± 1.16 ^ab^	13.54 ± 0.95 ^ab^	13.93 ± 0.56 ^a^	13.87 ± 0.95 ^a^	13.93 ± 0.73 ^a^
Titratable acidity (g/L, tartaric acid)	5.78 ± 0.76 ^a^	5.65 ± 0.50 ^a^	5.81 ± 0.88 ^a^	5.74 ± 0.44 ^a^	5.85 ± 0.72 ^a^	6.01 ± 0.70 ^a^
pH	3.83 ± 0.11 ^a^	3.70 ± 0.10 ^ab^	3.65 ± 0.11 ^b^	3.71 ± 0.16 ^ab^	3.70 ± 0.15 ^ab^	3.77 ± 0.16 ^a^
Total phenols (g/L, gallic acid)	2.52 ± 0.29 ^a^	2.79 ± 0.61 ^a^	2.84 ± 0.62 ^a^	2.83 ± 0.58 ^a^	2.84 ± 0.63 ^a^	2.94 ± 0.61 ^a^
Total anthocyanins (g/L)	0.05 ± 0.02 ^c^	0.08 ± 0.03 ^c^	0.13 ± 0.09 ^bc^	0.15 ± 0.06 ^bc^	0.21 ± 0.10 ^ab^	0.24 ± 0.05 ^a^
Total tannin (g/L)	3.14 ± 0.29 ^a^	3.16 ± 0.98 ^a^	3.03 ± 0.58 ^a^	3.34 ± 0.98 ^a^	3.29 ± 0.70 ^a^	3.30 ± 0.40 ^a^

Note: Different Latin letters indicate significant differences according to the HSD Tukey test (*p* < 0.05).

## Data Availability

Data are contained within the article or Supplementary Materials.

## Supplementary Materials

The following are available online at https://www.mdpi.com/article/10.3390/foods11010022/s1, Table S1: Details of the Wines in the Study from the Eastern Foot of Helan Mountain of Ningxia, California, and Bordeaux, Including the Regions of Origin Vintages, and the Oenological Parameters, Table S2: Quantitative standards, calibration curves, odor threshold for quantification, and odor description of volatile compounds, Table S3: Quantitative ion, quantitative standards, calibration curves, and quantitative limit of polyphenols, Table S4: Temperature and precipitation records, from March to September 2013–2018, in the six sub-regions of Ningxia, Figure S1: GC chromatogram of vine volatiles by HS–SPME of one sample, Figure S2: Chromatogram of polyphenols by LC–MS/MS of one sample.
